# Mask wearing as a prosocial behavior: Proposing and testing the moral norms activation model

**DOI:** 10.1371/journal.pone.0322921

**Published:** 2025-05-09

**Authors:** Monique M. Turner, Youjin Jang, Ruth Heo, Qijia Ye, Rachel Wade, Maria Knight Lapinski, Tai-Quan Peng

**Affiliations:** 1 Department of Communication, Michigan State University, East Lansing, Michigan, United States of America; 2 Lineberger Comprehensive Cancer Center, University of North Carolina at Chapel Hill, Chapel Hill, North Carolina, United States of America; 3 Annenberg School of Communication, University of Pennsylvania, Philadelphia, Pennsylvania, United States of America; 4 School of Communication, The Ohio State University, Columbus, Ohio, United States of America; 5 AgBioResearch, Michigan State University, East Lansing, Michigan, United States of America; Teikyo University - Hachioji Campus: Teikyo Daigaku - Hachioji Campus, JAPAN

## Abstract

The aim of this study was to develop and test a model of prosocial prevention behavior during COVID-19, termed the Moral Norms Activation Model (MNAM). This model examines how moral norms, influenced by awareness of consequences, predict prosocial prevention behaviors, such as mask-wearing, and the role of perceived severity and collective orientation as moderating factors. We conducted a survey during the early months of the COVID-19 pandemic with a nationally representative sample of U.S. adults (*N* = 8,778). The survey measured awareness of consequences, moral norms, anticipated guilt, perceived severity, collective orientation, and self-reported mask-wearing behavior. A series of regressions was used to test the proposed model and interactions. Findings supported the MNAM, demonstrating that awareness of consequences was a significant direct predictor of moral norms. These moral norms, in turn, predicted prosocial prevention behavior, mediated by anticipated guilt. The moderating effects of perceived severity and collective orientation were also significant, reinforcing the strength of the association between moral norms and behavior in individuals with high collective orientation and greater perceived severity. The results highlight the critical role of moral norms and anticipated guilt in promoting prosocial health behaviors during a collective health crisis. The MNAM provides a novel framework for understanding how individual psychological processes contribute to public health behaviors. These findings suggest that public health campaigns emphasizing moral responsibility and awareness of consequences could enhance compliance with preventive measures.

## Introduction

A unique characteristic of infectious disease is that individuals’ prevention and risk-taking behaviors affect the morbidity and mortality of others. It is impossible to “flatten the curve” if most of a given community does not engage in recommended preventive behaviors. For example, in the case of COVID-19 even those who are less susceptible to serious consequences of the virus (e.g., adolescents without comorbidities) were encouraged to engage in preventative actions like mask wearing and distancing for the benefit of their community. While traditional risk communication models focus on predictors of behavior such as perceived severity and susceptibility [1,2], we need to examine alternative predictors of behavior for a collective health risk context.

The norms activation model (NAM) proposes that prosocial behavior is best predicted by moral norms which, in turn, are predicted by awareness of consequences of one’s behavior and a sense of responsibility [3]. The current study extends the NAM by examining the roles of anticipated guilt and collective orientation on intention to engage in mask wearing during the COVID-19 pandemic. To date, the NAM has been tested in mainly environmental contexts (e.g., recycling, sustainable transportation) [4]; leaving the question as to whether the model fits in in infectious disease contexts. To test our theory, we used survey data from a nationally representative sample of U.S. adults that was collected during the summer of 2020.

## Literature review

### Prosocial behavior and moral norms

Prosocial behavior is defined as actions that are taken to benefit others and/or one’s community [5]. Based on this definition, prosocial prevention behavior includes risk-reducing or health promoting health behaviors that affect community members even if the behavior has little or no perceived personal benefit. For example, individuals may wear masks in public during COVID-19 because they do not want to be infected with the virus, but also, because they do not want to spread the virus to vulnerable others. That is, individuals who perceive low susceptibility and/or low personal severity of COVID-19 but still wear masks solely for the protection of others are engaging in prosocial prevention behavior.

One of the predictors of engaging in prosocial behaviors is moral norms [6]. Moral norms develop out of a sense of obligation people experience when achieving self-expectations, and these feelings of obligation develop into one’s personal normative standards [3]. Research has shown that moral norms can explain additional variance in behavioral intentions beyond common predictors such as attitude, subjective norms and perceived behavioral control [7]. Moral norms also predict prosocial behavior [8]. We draw from Schwartz’s NAM [3], which posits that the relationship between personal norms and behavior is affected by awareness of consequences and individual responsibility.

### Awareness of consequences of prosocial behavior

It has long been hypothesized that outcome expectation, expecting that one’s behavior will yield positive consequences [9], is predictive of engaging in that behavior. Outcome expectations (similar to response efficacy) is a critical variable in several communication theories that predict behavior (e.g., the extended parallel process model [EPPM]) [10]. For instance, Ort and Fahr found that promoting positive outcomes had a greater impact on Ebola vaccination attitudes and intention than emphasizing the threats of the disease [11]. We aim to extend this same idea, but in a context where the referent group is others as opposed to the self. That is, when people believe a behavior leads to positive consequences for others, their belief that it is “moral behavior” should increase. Adopting the verbiage of the NAM, we predict that as awareness of consequences (i.e., outcome expectation) increases, moral norms are more likely to activate and become salient.

Moreover, moral norms are correlated with feelings of responsibility. Specifically, when individuals believe that a prevention behavior is the “right” and “responsible” thing to do, the prosocial prevention behavior becomes more likely [5]. For example, Stern and colleagues found that in the context of environmental protection, people believe the government has a responsibility to act in the best interest of the general population, regardless of the government’s culpability in causing the problem [12]. We think this same logic can be applied to individual’s own behavior.

Taken together, we argue that awareness of consequences predicts moral norms, as moral norms encompass the notion of responsibility [13]. Schwartz was ambiguous about the role of awareness of consequences in his model. However, we suggest a mediated relationship such that:

**H1**: Awareness of consequences will have a positive association with moral norms.

**H2**: Moral norms will have a positive association with prosocial prevention behavior (in this case, self-reports of mask wearing).

Importantly, we must consider perceived severity as a potential moderator of this mediated relationship. The EPPM argues that when perceived threat is high (threat is partially comprised of severity) and perceived efficacy is high (partially comprised of response efficacy) then prevention behavior becomes more likely [2]. A meta-analysis exploring these relationships in experimental studies across a variety of health contexts found risk appraisal to have a greater effect on both intention and actual behavior when perceived severity was also increased [14]. In a similar vein, we argue that perceived severity of COVID-19 will interact with awareness of consequences to affect both moral norms and prosocial prevention behavior in the following moderated mediation relationship:

**H3**: Perceived severity of COVID-19 will moderate the relationship between awareness of consequences and mask wearing, such that when perceived severity is high and awareness of consequences is high, mask wearing will be at its highest.

**H4**: Perceived severity of COVID-19 will moderate the relationship between awareness of consequences and moral norms, such that when perceived severity is high and awareness of consequences is high, moral norms will be at its highest.

### Anticipated guilt

Guilt is a negatively valenced emotion arising when someone violates their moral standards or expects that a moral transgression could occur in the future [15]. According to cognitive appraisal theories, high self-controllability and high self-responsibility are two essential appraisals of guilt [15,16]. Brought about by self-reflection and self-evaluation, guilt is a self-conscious emotion that is typically accompanied with a desire to compensate for one’s transgressions, hopes of forgiveness, and wishes that one had not made the wrong acts [15,17,18]. When individuals experience guilt, they are motivated to alleviate the negative feelings by partaking in prosocial behavior [19].

Feelings of guilt can be caused by individuals simply thinking about the potential violation of moral rules of behavior [15,20]. Anticipated guilt, then, can occur when individuals imagine their own action or inaction leading to the harm of another person. Specifically, people experience greater guilt when violating socially approved rules that are linked to others compared to violating rules that they observe as common [21]. Therefore, anticipated guilt can act as a mechanism of social influence and, subsequently, alter people’s behavior [22].

Anticipated guilt is correlated with myriad behaviors, including charity donation [23], organ donation [24], emergency preparedness [25], recycling [26], and support for climate mitigation policy [27]. Rivis et al.’s meta-analysis showed anticipated emotion has significant effects on behavioral intention [7]. Thus, in the context of viral pandemics like COVID-19, those who feel stronger anticipated guilt should have stronger intentions to adopt preventive behavior elicited via any moral transgression or anticipated transgression [8].

**H5**: Anticipated guilt mediates the relationship between moral norms and prosocial prevention behavior.

### Collective orientation

Individuals who highly value the desires and needs of others score higher in collective orientation [28], which is related to the individualism-collectivism dimension [29–31]. People with stronger collectivistic orientations are more influenced by group norms due to their higher perceived importance on group goals and overall desire to maintain relational harmony [29–31]. Initially, collective orientation is known to be inherent from the cultural background (collective vs. individualistic) yet an increasing number of studies implies an individual characteristic [32].

Collective orientation has been found to strengthen perceived norms’ effect on both attitude about behavior as well as behavioral intention [33]. Particularly, more positive attitudes about behavior and stronger behavioral intention were reported by individuals who had stronger other-orientation compared to their more individualistic counterparts. A study by Cho et al. found collectivist orientation to negatively relate to COVID-19 spread and positively relate to attitudes and intentions surrounding prevention behavior [34]. Moreover, they explored this relationship via subjective norms such that collectivist individuals were more likely to be driven by the belief that others found COVID-19 prevention behaviors to be important. Other research suggests that participants may show more positive attitude and higher intention to follow norms to avoid feelings of guilt [19]. Furthermore, norms literature has demonstrated that more collective individuals experience greater levels of guilt when they violate norms [32,35]. Suresh and Walter explained that guilt effectively minimizes collective risks [36]. In the context of responding to impending risks like COVID-19, individuals who feel a strong moral obligation may experience increased guilt, particularly if they are concerned about the collective benefits and the potential harm they might impose on others. This relationship is tested in our sixth and final hypothesis:

**H6**: Collective orientation moderates the relationship between moral norms and anticipated guilt, such that when one is more collective, the relationship between moral norms and anticipated guilt will be stronger.

**Fig 1:**
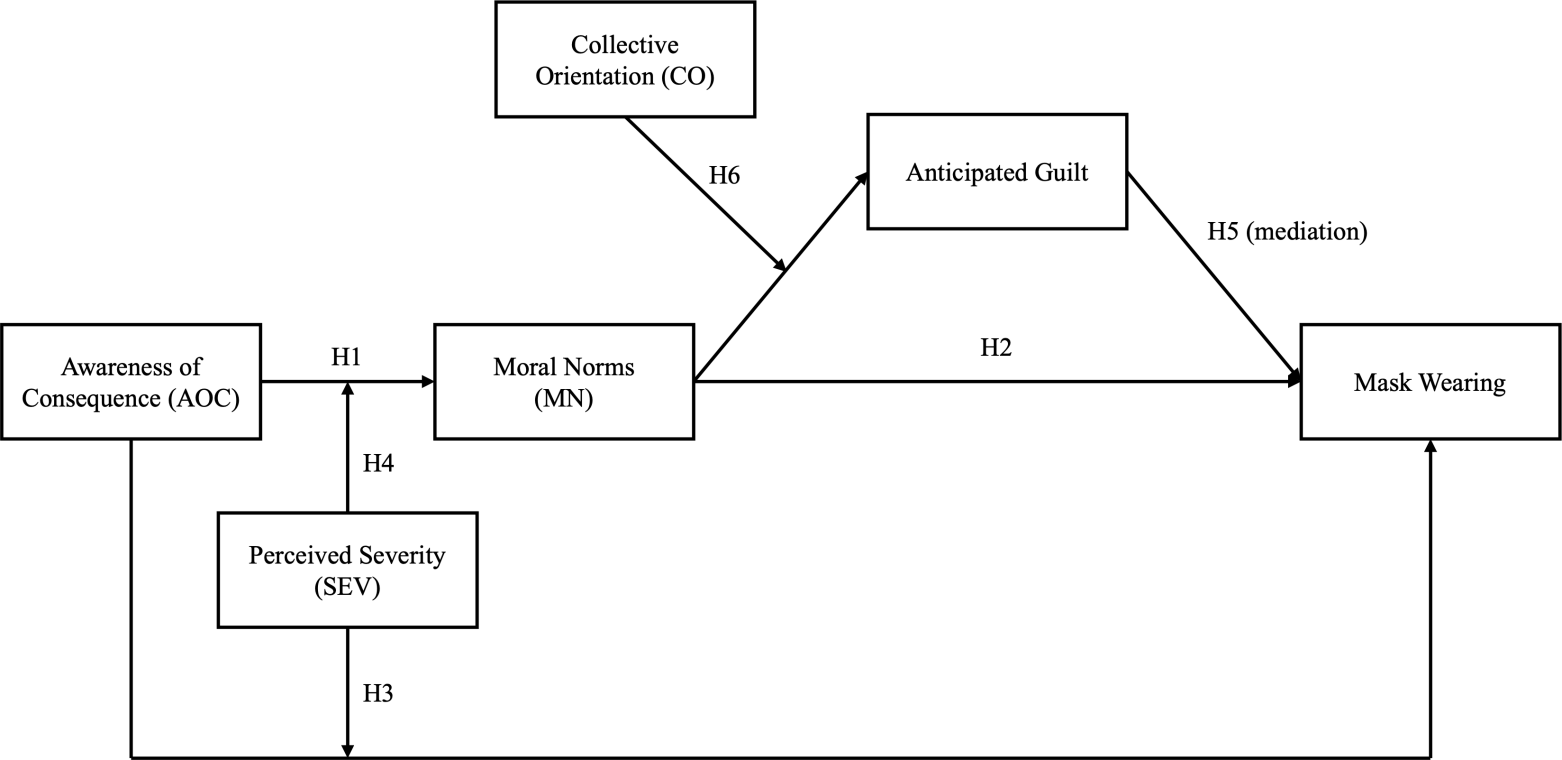
Theoretical model.

In sum, our hypotheses and research questions are depicted in Fig 1.

## Methods

### Sampling and participants

This study was part of a larger project. A rolling-cross sectional survey was conducted, using a national quota-based sample of adults aged 18 or older residing in the U.S. from July 6, 2020 to October 16, 2020. To achieve a margin of error of 5% and an unknown population size, it was necessary to collect at least *N* = 385. Given this study included mediated moderation analyses with multiple outcome variables, we decided to collect 9,000 observations, giving us an actual margin of error of 1%.

To control for the distinct federal, state, and local government policies over the pandemic influencing participants’ behaviors, we stratified by state. We sampled the top 10 states based on COVID-cases and selected five additional states that represented low and moderate level of COVID-19 prevalence (as of June, 2020), ensuring diversity in state mandates, public health policies and individual exposure to the pandemic. Employing quota-based sampling techniques, Qualtrics randomly selected approximately 500 adults every week for 17 weeks (totaling 8,778 valid responses), with quotas ensuring demographic alignment with the U.S. Census in terms of age, gender, race, and education level. Oversampling was conducted to ensure that all quotas were met. A total of 131,600 invitations were sent to pooled participants for a total of 32,489 responses. Participants who did not pass the attention check (*N* = 12,477) or did not complete at least 66% of the survey (*N* = 11,202) were removed from the sample. Additionally, responses were eliminated if they came in after the quota was filled or inappropriately recorded due to technical glitches. We excluded participants who believed that COVID-19 was a hoax (*n* = 2,424; 22%). After data cleaning, the final analytic sample size was 8,778, which provided sufficient power for detecting the hypothesized effects in the proposed moderated mediation model (Table 1).

**Table 1:** Demographic characteristics of participants.

	%		%
**Age**		**Education**	
18-34	32.5	Less than high school	3.5
35-55	34.8	High school graduate	26.7
55 +	32.7	Some College but no degree	19.8
**Biological Sex**		Associate degree in college	10.8
Female	51.3	Bachelor’s degree in college	23.2
Male	48.7	Master’s degree	12.3
**Household Income**		Doctoral degree	1.5
<30k	28.8	Professional degree	2.1
30k ≦ and < 60k	27.8	**Political Party**	
60k ≦ and < 90k	18.3	Republican	28.8
90k ≦	25.1	Democrat	36.0
**Race**		Other	35.2
White	70.4	**Diagnosed with COVID (Self)**	
Black	16.4	Yes	0.2
American Indian	1.9	No	97.8
Asian	6.8	**Diagnosed with COVID (Others)**	
Native Hawaiian	0.5	Yes	39.1
Others	4.0	No	60.9

### Procedure

This study was deemed exempt by the Human Subjects Review Board at Michigan State University (MSU) (Study ID: 00004287). The study involved an online survey, and participants were provided with an informed consent form at the beginning of the survey. Consent was obtained electronically, where participants indicated their agreement to participate by clicking the “Next” button to proceed with the survey. Upon invitation, participants directed to the survey were asked about their demographics, psychosocial constructs (moral norms, anticipated guilt, severity, collectivism), and prevention behavior (mask wearing). The survey also included an attention check, presented randomly, which was passed or failed by entering a prompted number.

Data were collected weekly for 17 weeks, using the quota sampling procedure delivered by Qualtrics. Survey items were organized into blocks, including general demographics, psychosocial constructs, and prevention behavior (Table 2). All the descriptive statistics of variables are reported in Table 3. To control order effects, blocks and questions within the individual blocks were randomized, except for demographic questions.

**Table 2:** Measurements.

Constructs	Items	*ɑ*
Awareness of consequences	1. I am less likely to spread COVID-19 to others if I engage [or continue to] in mask wearing. 2. I can help slow the spread of COVID-19 in society if I engage [or continue to] in mask wearing.	.85
Perceived severity	1. COVID-19 is a serious health threat 2. There are severe health repercussions from getting COVID-19	.70^**^
Collective orientation	1. I would do what would help people around me, even if I didn’t like doing it. 2. I usually sacrifice my self-interest for the benefit of others around me. 3. My happiness depends very much on the happiness of others around me. 4. It is important for me to maintain harmony with people around me. 5. I do what is right for people around me, even if it is difficult.	.82
Moral norms	1. I believe that I should wear a mask if I am out in public. 2. I personally believe that it is my moral responsibility to wear a mask when I am out in public. 3. I believe that wearing a mask in public is the right thing to do.	.95
Anticipated Guilt	1. I would feel guilty if.... I went out in public without a mask. 2. I would feel guilty if.... I was in public and didn’t bring a mask.	.78^**^
Masking behavior intention	1. Wear a mask when I am in a public place (like a restaurant or park). 2. Wear a mask when I exercise at the gym. 3. Wear a mask when exercising outdoors (walking, riding a bike or running for example). 4. Wear a mask when I am out.	.76
Perceived susceptibility	1. How likely is it that you will get COVID-19 at some point in the future? 2. When I think carefully about my lifestyle, it does seem possible that I could get COVID-19	.63^**^
Self-efficacy	1. It is easy for me to wear a mask when I am in public 2. I am confident that I can wear a mask when I go out of my house	.69^**^

*Notes*. When the number of items is less than 3, the correlation coefficient was calculated. ^*^ *p* < .05, ^**^ *p* < .01

**Table 3:** Descriptive statistics and correlations between variables.

Variables	1	2	3	4	5	6	7	8
1.Moral Norms	⎯							
2.Severity	.61^**^	⎯						
3.Susceptibility	.18^**^	.27^**^	⎯					
4.Mask wearing	.59^**^	.50^**^	.17^**^	⎯				
5.Awareness of consequences	.69^**^	.63^**^	.15^**^	.59^**^	⎯			
6.Anticipated guilt	.62^**^	.52^**^	.14^**^	.47^**^	.57^**^	⎯		
7.Self-efficacy	.67^**^	.55^**^	.15^**^	.60^**^	.71^**^	.52^**^	⎯	
8.Collective orientation	.36^**^	.31^**^	.13^**^	.28^**^	.30^**^	.33^**^	.28^**^	⎯
*Mean*	6.11	78.60	46.06	71.25	77.15	5.32	81.40	5.44
*SD*	1.52	25.59	27.10	27.95	28.04	1.87	25.74	1.04
*Minimum*	1	0	0	0	0	1	0	1
*Maximum*	7	100	100	100	100	7	100	7

** *p* < .01.

### Measurement

#### Covariates.

Covariates included perceived susceptibility and self-efficacy given their known relationships with prevention behavior [2]. Following Tabachnick and Fidell [37], we selected covariates based on their evidence-based substantial association with the dependent variable, prioritizing those with the strongest relationships to maximize statistical power and avoid redundancy when they were correlated with other independent variables.

#### Awareness of consequences.

Awareness of the consequences of mask wearing was measured with a slider from 0 (“none” or 0%) to 100 (“completely” or 100%). Two items were used including “I am less likely to spread COVID-19 to others if I wear (or continue to) a mask in public” [5].

#### Perceived severity.

Perceived severity about COVID-19 was measured with a slider from 0 (“none”) to 100 (“completely”). Two items were used including “COVID-19 is a serious health threat” [2].

#### Moral norms.

Moral norms were measured with a 7-point Likert scale (from 1 = strongly agree to 7 = strongly disagree; later reverse coded). Three items were used including “I believe that wearing a mask in public is the right thing to do” [38].

#### Anticipated guilt.

Anticipated guilt was measured with a slider from 0 (“none” or 0%) to 100 (“completely,” or 100%) and 7-point Likert scale. Two items were used including “I would feel guilty if... I went out in public without a mask” [39].

#### Collective orientation.

Collective orientation was measured with a 7-point Likert scale (from 1 = strongly agree to 7 = strongly disagree). Five items were used, including “I would do what would help people around me, even if I didn’t like doing it” [28,33].

#### Prosocial prevention behavior.

Participants were asked to indicate the extent to which they would wear a mask with different rating scales such as a slide from 0 (“none” or 0%) to 100 (“completely” or 100%). Four items were used such as “wear a mask when I am out” [40].

### Analyses

To test the hypotheses, we first examined bivariate correlations among all variables in our theoretical model (Table 2). Next, we conducted a series of regressions using SPSS Version 23 with the purpose of assessing the ability of the independent variable and each mediator to predict mask wearing, controlling for demographics, perceived susceptibility, and self-efficacy.

## Results

A simple regression was performed to test hypothesis 1. The regression analysis revealed that the model significantly predicted moral norms, *F*(1, 8776) = 8096.12, p < .001, *R*^*2*^ = .69. Awareness of consequences was found to be positively related to moral norms, *B* = .04, *SE* = .00, *p* < .001. This indicates that an increase in awareness of consequences was associated with an increase in perceived moral norms, which was consistent with hypothesis 1.

A multiple regression was conducted to test hypothesis 2. The regression analysis showed that the model significantly predicted mask wearing behaviors, *F(*3, 8774) = 2152.13, *p* < .001, *R*^*2*^* *= .65. While controlling covariates (i.e., perceived susceptibility and self-efficacy), the association between moral norms and mask wearing behaviors was statistically significant, *B* = 6.24, *SE* = .20, *p* < .001. That is, when individuals perceived stronger moral norms, they were more likely to wear masks. Therefore, the data were consistent with hypothesis 2.

**Fig 2:**
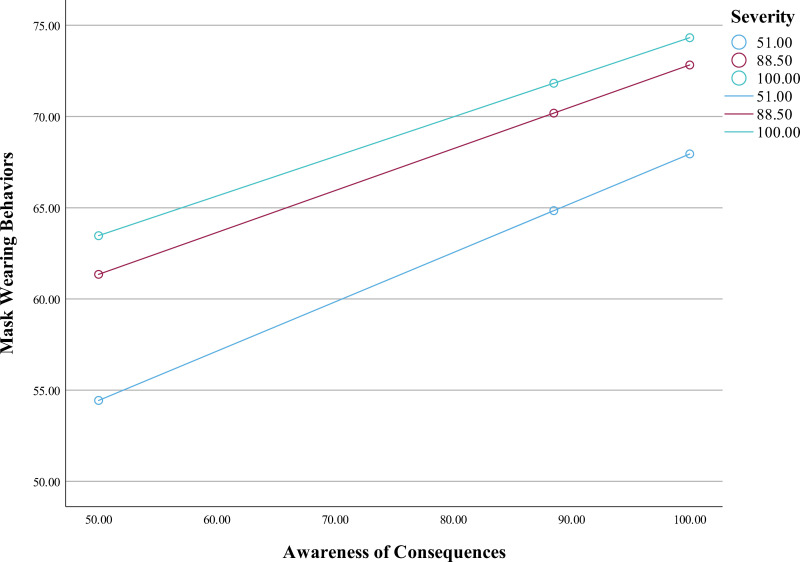
The moderating effect of perceived severity on mask wearing behaviors.

To test hypothesis 3, SPSS PROCESS was used (Model 1) [41]. The model significantly predicted mask wearing behaviors, *F*(5, 8772) = 1311.17, *p* < .001, *R*^*2*^ = .43. While holding the covariates constant, the main associations between awareness of consequences (*B* = .33, *SE* = .02, *p* < .001) and mask wearing behaviors, and between perceive severity (*B* = .34, *SE* = .01, *p* < .001) and mask wearing behaviors were both statistically significant. As predicted, the interaction term (awareness of consequences and perceived severity) was also found to be statistically significant (Tables 4 and 5). Mask wearing was at its highest when perceived severity was high and awareness of consequences was high, *B* = -.001, *SE* = .0003, *p* < .001 (Fig 2). Therefore, the data were consistent with hypothesis 3.

**Table 4:** Regression with awareness of consequences and severity.

IVs	*B*	*SE*	*t*	*p*
DV: Moral norms				
Constant	1.58	.05	28.80	<.001
Awareness of Consequences	.05	.00	51.38	<.001
Severity	.04	.00	39.28	<.001
AOC * Severity	-.00	.00	-25.75	<.001
DV: Mask wearing behaviors				
Constant	-1.08	1.18	-.91	.36
Awareness of Consequences	.33	.02	14.72	<.001
Severity	.24	.02	11.36	<.001
AOC * Severity	-.00	.00	-3.98	<.001
Susceptibility	.04	.01	4.46	<.001
Self-efficacy	.34	.01	26.92	<.001

*Notes.* For model 1 (DV: Moral norms), *F*(3, 8774) = 3775.99, *p* < .001, *R*^*2*^ = .56. For model 2 (DV: Mask wearing behaviors), *F*(5, 8772) = 1311.17, *p* < .001, *R*^*2*^ = .43.

**Table 5:** Conditional effect of awareness of consequence at values of severity on moral norms.

Level of Severity	*Effect*	*SE*	*t*	*p*	*Confidence Interval*	
					*Lower Level*	*Upper Level*
DV: Moral norms						
51.00	.03	.00	61.79	<.001	.03	.03
88.50	.02	.00	33.32	<.001	.02	.02
100.00	.02	.00	23.24	<.001	.01	.02
DV: Mask wearing behaviors						
51.00	.27	.01	20.50	<.001	.24	.30
88.50	.23	.01	16.72	<.001	.20	.26
100.00	.22	.02	14.17	<.001	.19	.25

**Fig 3:**
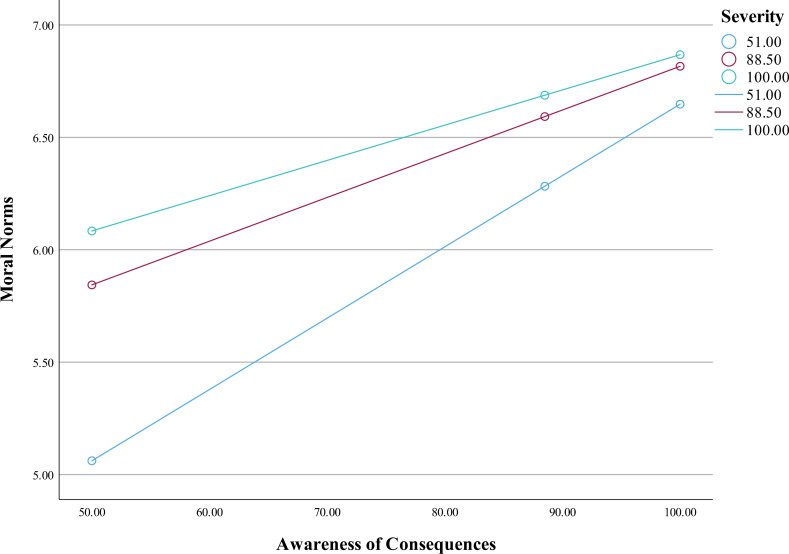
The moderating effect of perceived severity on moral norms.

To test hypothesis 4, SPSS PROCESS was used (Model 1) [41]. The model significantly predicted moral norms, *F*(3, 8697) = 3393.06, *p* < .001, *R*^*2*^ = .54. The main associations between awareness of consequences and moral norms (*B* = .05, *SE* = .001, *p* < .001), and between perceived severity and moral norms (*B* = .04, *SE* = .001*, p* < .001) were statistically significant. The interaction effect of awareness of consequences and perceived severity was statistically significant (Tables 4 and 5); moral norms were highest when perceived severity was high and awareness of consequence was high, *B* = -.0003, *SE* = .000, *p* < .001 (Fig 3). Thus, the data were consistent with hypothesis 4.

To test hypothesis 5 and 6, SPSS PROCESS was again used (Model 7) [41]. The moderated mediation model significantly predicted mask wearing, *F*(5, 8695) = 1175.27, *p* < .001, *R*^2^ = .40. The main association between moral norms and anticipated guilt was statistically significant, *B* = .45, SE = .04, *p* < .001, while controlling covariates. The main association between moral norms and mask wearing behaviors was also statistically significant, *B* = 5.24, SE = .23, *p* < .001, as well as the effect of anticipated guilt on mask wearing behaviors, *B* = 1.62, SE = .16, *p* < .001 (Table 6). Regardless of the level of collective orientation, the indirect effect of moral norms on mask wearing behaviors via anticipated guilt was statistically significant (Table 7). Therefore, the data were consistent with hypothesis 5.

**Table 6:** Moderated mediation effect of anticipated guilt and collective orientation.

IVs	*b*	*SE*	*t*	*p*
DV: Anticipated guilt				
Constant	.35	.22	1.62	.10
Moral norms	.42	.04	10.97	<.001
Collective orientation	.05	.05	1.10	.27
Moral norms * Collective orientation	.03	.01	3.70	<.001
Susceptibility	.00	.00	2.37	.02
Self-efficacy	.01	.00	17.33	<.001
DV: Mask wearing behaviors				
Constant	-7.80	.97	-8.02	<.001
Moral norms	5.26	.22	23.77	<.001
Anticipated guilt	1.65	.16	10.56	<.001
Susceptibility	.05	.01	6.35	<.001
Self-efficacy	.37	.01	30.86	<.001

*Notes.* For the model 1 (DV: anticipated guilt), *F*(5, 8772) = 1270.30, *p* < .001, *R*^*2*^ = .42. For the model 2 (DV: mask wearing behaviors), *F*(4, 8773) = 1662.29, *p* < .001, *R*^*2*^ = .43.

**Table 7:** Conditional effects of moral norms at value of collective orientation.

Level of collective orientation	*Effect*	*SE*	*Confidence Interval*	
			*Lower Level*	*Upper Level*
DV: Anticipated guilt				
4.40	.54	.01	.52	.57
5.60	.58	.02	.55	.61
6.40	.60	.02	.56	.63
DV: Mask wearing behaviors				
4.40	.90	.10	.71	1.09
5.60	.95	.11	.75	1.17
6.40	.99	.11	.77	1.21

Notes. SE and confidence interval for mask wearing behaviors were calculated by bootstrapping (*n* = 5000). The index of moderated mediation for collective orientation is.05, SE = .02, 95% CI [.02,.07]. The direct effect of moral norms on mask wearing behaviors is 5.26, SE = .22, *t* = 23.77, *p* < .001.

**Fig 4:**
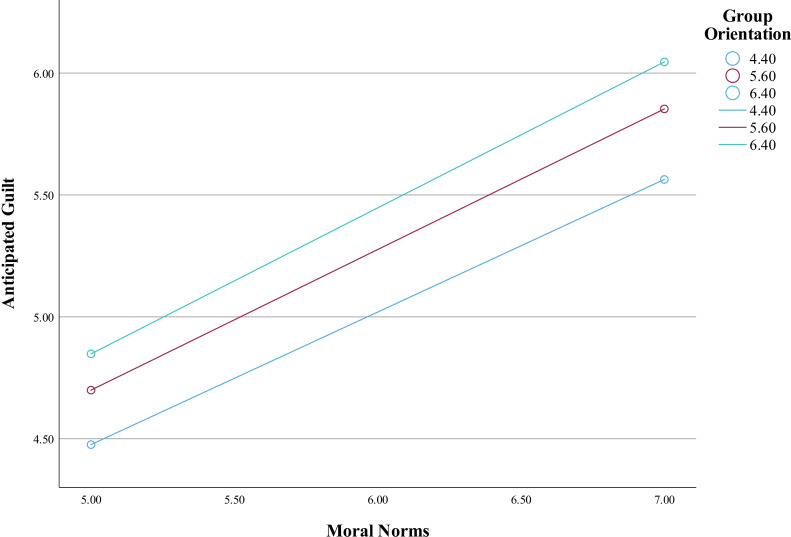
The moderating effect of group orientation on anticipated guilt.

The association between collective orientation and anticipated guilt was not statistically significant, *B* = .08, *SE *= .05, *p* > .05. But the interaction effect of collective orientation and moral norms on anticipated guilt was found to be statically significant, *B* = .03, *SE* = .01, *p* < .01 (Table 6, Fig 4). That is, collective orientation was found to strengthen the relationship between moral norms and anticipated guilt. Thus, the data were consistent with hypothesis 6.

## Discussion

The Moral Norms Activation Model (MNAM) expands on existing health behavior models by integrating moral and collective motivation factors, which are often overlooked in traditional risk-based models. While many post-COVID prediction models have adapted frameworks such as the Health Belief Model (HBM) [42] or the Theory of Planned Behavior (TPB) [43] to explain compliance with preventive measures, these models primarily focus on individual risk perceptions, attitudes, and self-efficacy as determinants of behavior. In contrast, MNAM emphasizes the role of moral norms, collective orientation and anticipated guilt as key psychological mechanisms driving prosocial prevention behaviors. This distinction is particularly important for behaviors like mask-wearing, which are not solely based on self-protection but also involve considerations for community well-being. By demonstrating that moral norms can be activated through awareness of consequences and that their influence on behavior is amplified by collective orientation, our model highlights a novel pathway for designing more effective public health interventions. These findings suggest that strategies aimed at increasing adherence to prevention behaviors should not only emphasize personal risk but also strengthen moral responsibility and collective concerns.

Our findings showed that perceived severity and awareness of consequences were related to moral norms, which were also related to anticipated guilt and mask-wearing behaviors, even after controlling for perceived susceptibility and self-efficacy, as these factors related to COVID-19 varied across age groups and those with certain comorbidities. In other words, individuals who were aware of the seriousness of COVID-19 and how wearing masks could alleviate its spread were more likely to develop moral norms. These moral norms, in turn, were positively related to stronger anticipated guilt when not wearing masks and to more frequent mask-wearing behaviors. This indicates that infectious disease crises entail much more than personal risk perceptions, although traditional risk theories tend to predict prevention behaviors as a consequence of personal risk perceptions (including personal severity and susceptibility) and efficacy perception [2,44]. Given this, it is vital that moral norms are activated by enhancing awareness of consequences and that anticipated guilt is made salient to heighten one’s probability of engaging in prosocial behaviors, especially when one perceives lower susceptibility and a lower likelihood of severe consequences from infectious diseases.

In the context of infectious disease, messaging should emphasize the potential positive outcomes for the community of engaging in protective behaviors, especially for those who are less vulnerable to the disease. For example, in the COVID-19 case, this could include highlighting how wearing a mask can lead to decreased transmission of the disease and reduce the impact on vulnerable populations. Given that stronger moral norms are associated with higher mask-wearing behaviors, public health campaigns should frame mask-wearing as a moral obligation. Messages could focus on the ethical responsibility individuals have to protect their communities, emphasizing that wearing a mask is the “right thing to do.”

Based on our findings, this strategy would be more effective for those who have a stronger collective orientation, as salient moral norms would result in stronger anticipated guilt when accompanied by a stronger collective orientation. However, this strategy should be used cautiously, as enhancing guilt often involves evoking anger or distress [25,45], which might be counter-productive in this case. It is important to balance motivating prosocial behaviors and avoiding potential negative responses.

Our findings also indicated that it is important to understand the interplay between personal risk perceptions and social motivation factors (e.g., moral norms and collective orientation) for infectious diseases or risks that require collective actions and prosocial behaviors. Exploring how combining personal and social motivators can create more comprehensive public health strategies becomes more crucial when designing messages for different communities, as cultural differences might affect the perception of moral norms [46] and the effectiveness of guilt-based messaging [47]. Interestingly, our findings revealed that the mean collective orientation score (*M* = 5.44, *SD* = 1.04) was relatively high, despite the U.S. being generally viewed as an individualistic culture. One plausible explanation is that the survey context—focused entirely on COVID-19—may have primed participants to think in terms of collective action. Research suggests that U.S. political leaders actively mobilized collective intentionality during the pandemic to foster prosocial behaviors, encouraging citizens to act for the common good regardless of partisan differences [48]. This emphasis on community and collective responsibility may have heightened participants’ perceptions of collective orientation during the time of data collection. Additionally, since all survey questions were framed in the context of COVID-19, it is possible that respondents were influenced by contextual cues, leading them to report stronger collective tendencies than they might in other contexts. Future research should examine how such situational influences shape self-reported collective orientation and whether these effects persist beyond crisis situations.

Beyond mask-wearing, the MNAM may apply to other public health behaviors that involve both individual risk reduction and collective protection. One example is hand hygiene, which plays a crucial role in preventing the spread of infectious diseases. Like mask-wearing, handwashing can be framed as a moral obligation, particularly in contexts where people interact with vulnerable individuals (e.g., hospitals, schools, public spaces). Future research could examine how awareness of consequences and moral norms influence consistent hand hygiene practices and whether anticipated guilt plays a role in adherence. Additionally, MNAM may be useful for understanding behaviors beyond infectious disease prevention, such as climate-friendly actions, where moral responsibility and collective orientation influence engagement in prosocial behaviors. These areas offer promising directions for further expanding MNAM’s applicability across various domains requiring collective action.

### Limitations and future research direction

This study is not without limitation. One limitation of the current study is its inability to establish causal relationships between the variables. As the research is correlational, it cannot definitively determine that moral norms, perceived severity, and awareness of consequences directly cause changes in prosocial behaviors. To address this, future research should focus on conducting controlled experiments. By manipulating key variables such as moral norms and awareness of consequences in a controlled environment, researchers can observe their direct effects on prosocial behaviors, thereby establishing a causal link.

Another limitation involves the scope of the outcomes measured in the study. This research primarily focused on the awareness of positive outcomes from engaging in prosocial behaviors. However, it remains unclear how the awareness of negative outcomes from not engaging in these behaviors might influence moral norms and subsequent actions. Future studies should compare the effects of awareness of positive outcomes with awareness of negative outcomes to provide a more comprehensive understanding of how different types of awareness impact prosocial behaviors.

Furthermore, the MNAM needs to be tested in various cultural contexts to assess its applicability and robustness. The study’s findings may not generalize across different cultures, where moral norms and collective orientation might function differently. There are numerous situations where individuals with lower personal risk are still encouraged to engage in prevention behaviors for the benefit of others, such as flu prevention, antibiotic resistance stewardship, and the disclosure of HIV status. Applying the MNAM to these contexts can help validate the model and understand its effectiveness in diverse scenarios.

## Conclusion

Our study developed the MNAM by extending the NAM, emphasizing moral norms’ role in prosocial behavior. We found that perceived severity and awareness of consequences significantly influence moral norms and mask-wearing behaviors, especially within a collective context. Our research indicates that infectious disease crises require more than personal risk perceptions. Traditional risk theories, focusing on personal severity, susceptibility, and efficacy, do not fully capture the complexity of motivating prosocial behaviors. Enhancing awareness of consequences and making anticipated guilt salient can significantly boost prosocial behaviors, even when personal risk is perceived as low.
